# Proton pump inhibitor induced hypocalcemia presenting with carpopedal spasm- a rare case report from Nepal

**DOI:** 10.1186/s12245-026-01239-5

**Published:** 2026-04-30

**Authors:** Nabin Pahari, Mukesh Pahari, Sagun Ghimire, Anusha Regmi, Prabhat Kaphle, Achyoot Sharan Koirala

**Affiliations:** 1 Department of Intensive Care, Mercy City Hospital, Rupandehi, Nepal; 2 Devdaha Medical College and Research Institute, Rupandehi, Nepal; 3 Department of Neurosurgery, Kist Medical College and Teaching Hospital, Kathmandu, Nepal; 4 University of New England, Sydney, Australia; 5 National Health Action Force Nepal, Kathmandu, Nepal; 6 Department of Medicine, College of Medical Science, Chitwan, Nepal

**Keywords:** Carpopedal spasm, Case report, Electrolyte imbalance, Hypocalcaemia, Nepal, Tetany

## Abstract

**Background:**

A frequent electrolyte imbalance, hypocalcaemia can cause anything from asymptomatic symptoms to potentially fatal neuromuscular and cardiac symptoms. Tetany, carpopedal spasm, and ECG abnormalities such QT prolongation can result from acute drops in serum calcium. Long-term usage of proton pump inhibitors (PPIs) is frequently disregarded as a reversible etiology, despite the fact that drugs are known causes. To avoid major issues and guarantee timely treatment, early detection is essential.

**Case presentation:**

Acute painful carpopedal spasms and perioral numbness were reported by a 35-year-old man who had previously been in good health. He had a history of using pantoprazole on his own for seven years without a doctor's supervision. Electrocardiography showed QT prolongation, while a clinical examination showed a positive Trousseau’s sign. Laboratory tests revealed normal levels of magnesium and vitamin D but severe hypocalcaemia (serum calcium 4.5 mg/dL). In addition to stopping PPI treatment, the patient was treated with intravenous calcium gluconate and oral calcium supplements. His symptoms quickly went away, and during follow-up, his serum calcium returned to normal.

**Conclusion:**

This case demonstrates that long-term PPI usage is an uncommon but significant reversible cause of severe hypocalcaemia that manifests as abrupt neuromuscular symptoms. When a patient has unexplained tetany or carpopedal spasms, clinicians should remain highly suspicious. To avoid potentially fatal consequences and guarantee positive results, early detection, stopping the offending drug, and prompt calcium replacement are crucial.

## Introduction

Calcium is a mineral the body must have so that cells in the nervous, cardiovascular and musculoskeletal systems work properly. Inside the cell, calcium acts as a second messenger - it triggers the electrical spike that lets nerves fire, it lets vesicles release neurotransmitter plus it switches enzymes on or off [1]. The ionized form of this mineral regulates many microscopic cellular processes. It activates protein kinases, promotes the phosphorylation of enzymes, and enables cells to respond to hormones such as epinephrine, glucagon, vasopressin, secretin, and cholecystokinin [2, 3]. Calcium balance in the blood is kept steady by four factors - parathyroid hormone, calcitonin, the active form of vitamin D (1α, 25dihydroxyvitamin D) and the amounts of calcium and phosphate already present in the serum [4]. Calcitonin reduces calcium - blocking the work of osteoclasts [5]. Hypocalcemia is an electrolyte disorder - it exists when the corrected total calcium in serum falls below 8.5 mg/dL or when the uncorrected value drops under 2.12 mmol/L [6]. Proton pump inhibitors belong to the drug classes most often prescribed in primary care. They represent a major advance against acid peptic disease. Acute hypocalcemia shows itself as neuromuscular irritability that ends in tetany. The signs run from mild paraesthesia to severe carpopedal spasm, laryngospasm or seizures.

## Case presentation

A​‍​‍​‌‍​‍‌​‍​‌‍​‍‌ 35-year-old male, generally healthy, presented to the emergency department with muscle spasms that were painful in the hands and had started about 18 h before his arrival. At first, he only felt some strange peri-oral numbness, but soon spasms began to happen in each of his hands. He did not have nausea, vomiting, abdominal pain, diarrhea, vomiting blood, or black stools. Moreover, he did not have a fever. Weakness and fatigue appeared later, and the spasticity became stronger with the passage of time. The man explained that the spasms were claw-like gripping contractions which could last for several minutes and the release would be done spontaneously, and the spasms would come again. When the spasms became more frequent, more intense, and finally, he could not move and, therefore, had to seek medical help. He denied any prior history of thyroid, parathyroid, or neck surgery, neck irradiation, autoimmune disease, bowel disease, chronic kidney disease, or known endocrine disorder. There was no history suggestive of autoimmune polyglandular syndrome, malignancy, or paraneoplastic disorder, significant weight loss, or other features suggestive of overt malnutrition.

On further history, the patient reported regular self-administration of pantoprazole 40 mg once daily for approximately 7 years for recurrent dyspeptic symptoms without prior formal medical evaluation. No prior upper gastrointestinal endoscopy or formal evaluation for dyspepsia, GERD, or peptic ulcer disease had been performed, so the appropriateness of prolonged PPI therapy could not be confirmed.

He denied infection recently, and high caffeine and alcohol intake recently. He also denied diuretic, laxative, herbal remedy, or other medication use. There was no family history of parathyroid or thyroid disease. He was a non-smoker and drank alcohol only ‍​‌‍​‍‌​‍​‌‍​‍‌occasionally. This was the first episode of symptomatic hypocalcemia, and the patient denied any prior milder episodes or subtle symptoms such as tingling, cramps, or numbness before this presentation though he may have experienced occasional nonspecific symptoms such as intermittent fatigue and mild muscle cramps/paresthesia prior to this presentation.

No history of fragility fractures, chronic bone pain, or prior diagnosis of metabolic bone disease.

He denied recent dietary restriction, chronic poor intake, significant weight loss, prolonged fasting, or symptoms suggestive of overt malnutrition. His nutritional history did not suggest clinically significant malnourishment. There was no recent travel, and the patient did not come into contact with anyone who was ill, nor did he eat any contaminated food or drink contaminated water. In addition, he denied excessive physical activity, hyperventilation, and anxiety attacks, as well as trauma, seizures, and loss of consciousness.

**Fig. 1:**
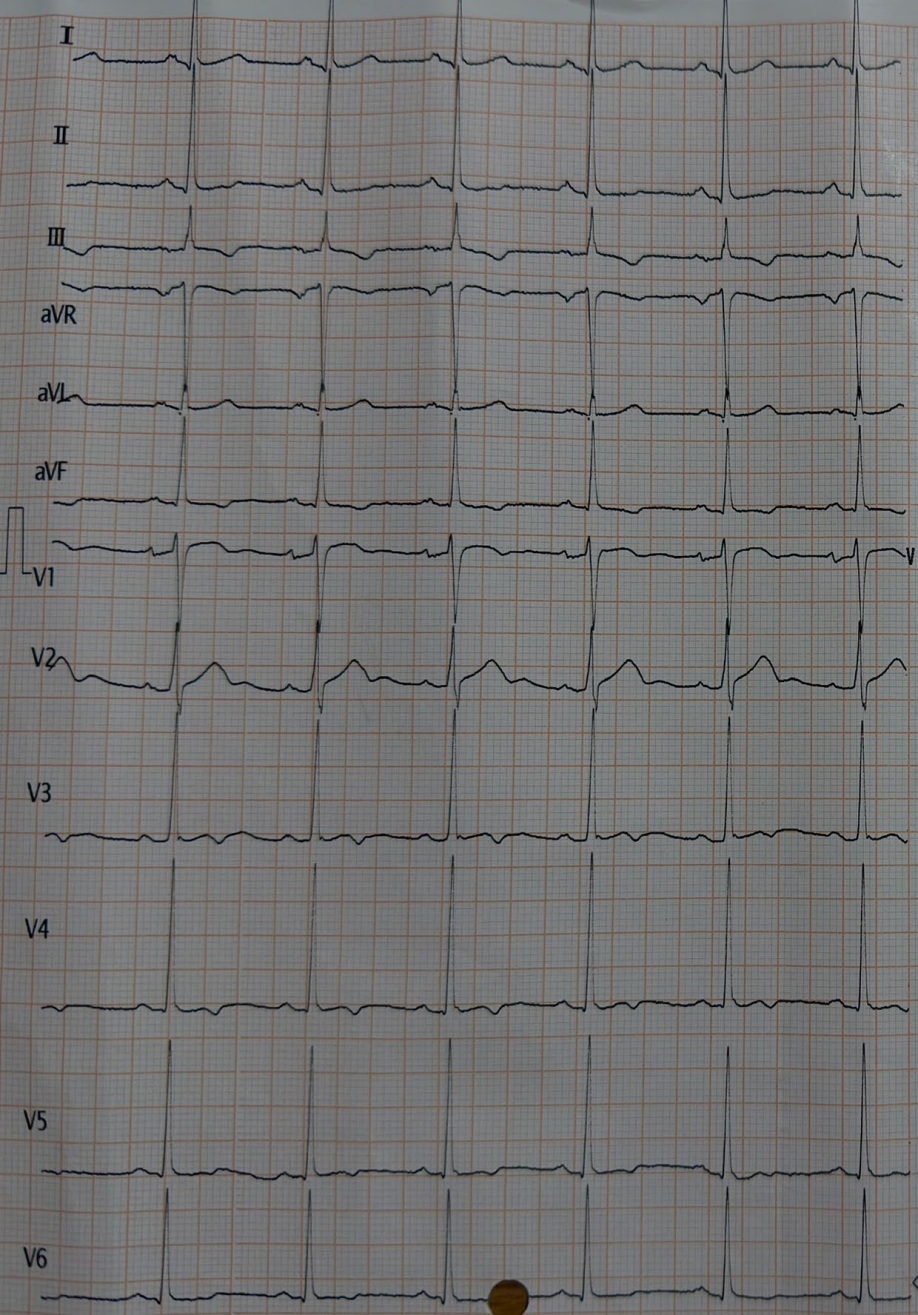
An electrocardiogram (ECG) showed QT interval prolongation consistent with hypocalcemia

**Fig. 2:**
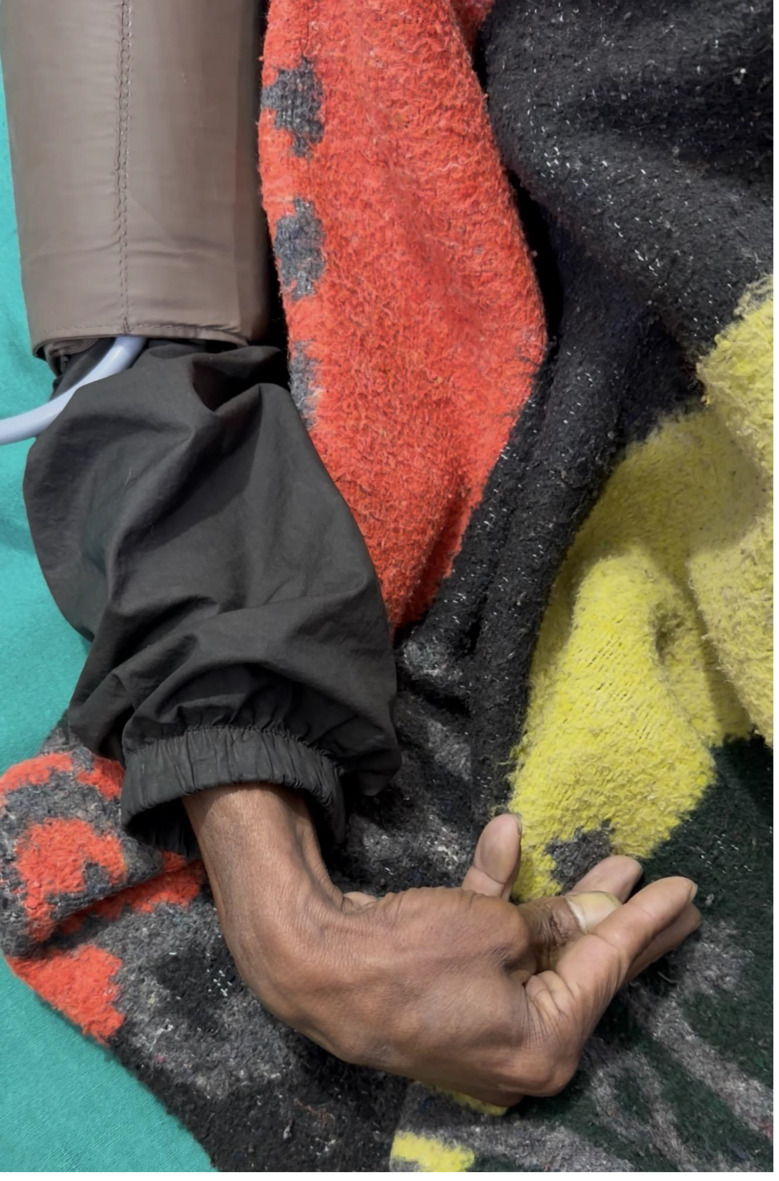
Carpopedal spasm (Trousseau’s sign)

The patient was brought to the emergency department, and he was conscious but very distraught. His heart and blood pressure were within normal limits and he was not dehydrated. His 12 leads ECG showed long QT prolongation shown in Fig. 1. On the neurologic examination, the patient demonstrated neuromuscular irritability with Trousseau’s signs being positive shown in Fig. 2. He also reported recurrent cramping in his lower limbs, though no overt tetany was observed.

The physical examination of the cardiac, respiratory, and abdominal systems did not disclose any abnormalities.

The story of prolonged use of proton pump inhibitors, neuromuscular symptoms, and physical signs pointed to us first to the suspicion of an acute electrolyte imbalance, most likely hypocalcemia caused by PPI overuse and impaired calcium absorption. This prompted us to order further laboratory tests to confirm the diagnosis.

## Treatment protocol

### Management and treatment

The patient was admitted for close monitoring so that symptomatic hypocalcaemia could be spotted early and corrected at once and so that any related electrolyte disturbances could be found. During hospitalization, pantoprazole was discontinued. The first aim of treatment was to stop neuromuscular irritability quickly plus to prevent further problems.

Treatment began with intravenous calcium where the patient received 10 mL of 10% calcium gluconate, diluted in 100 mL of 0.9% normal saline, given slowly over 10–15 min while the heart rate and rhythm was monitored on a cardiac monitor. The same dose was repeated every 6–8 h, guided by the patient’s clinical response and by repeated ionised calcium measurements, until the carpopedal spasms stopped but also serum calcium levels began to rise.

Serum magnesium was within the normal range - magnesium supplements were not needed - hypomagnesaemia had been ruled out as a cause of the hypocalcaemia. Vitamin D levels were also normal and no loading or maintenance vitamin D was given during the stay.

Once the patient was stable also able to swallow, he was switched to oral calcium carbonate 1–2 g/day to keep serum calcium normal and to stop hypocalcaemia reoccurrence.

During hospitalization, the patient was kept at Intensive Care Unit with closely monitored with serum electrolytes tests and a continuous electrocardiographic surveillance. The dose of calcium was adjusted based on the patient’s biochemical response and clinical condition. After a few hours of starting the treatment, the patient was totally free of muscle spasms.

Serum calcium levels became stable and were maintained at the normal range during the next 24–48 h. Patient blood investigations are shown in Table 1.

## Investigations

Initially laboratory test revealed significant hypocalcemia. The Magnesium levels were borderline low, meanwhile other electrolytes and renal function tests were normal. Arterial blood gas (ABG) was within normal limits, with no evidence of acid-base disturbance.

**Table 1:** Laboratory investigations

Laboratory Parameter	Patient Value during arrival	Patient Value during discharge	Laboratory Reference Range
Total Serum Calcium	4.5 mg/dl	9.1 mg/dl	8.5–10.5 mg/dl
Serum Albumin	3.4 g/dl	4.6 mg/dl	4.4–5.2 mg/dl
Serum Phosphate	2.0 mg/dl	3.1 mg/dl	2.5–4.5 mg/dl
Serum Magnesium	1.8 mg/dl	1.8 mg/dl	1.7–2.5 mg/dl
Parathyroid Hormone (PTH)	9 pg/mL	50 pg/mL	10–65 pg/mL
25(OH) Vitamin D	22 mg/dl	22 mg/dl	20–50 ng/mL
Urinary Calcium	60 mg/dl	120 mg/dl	100–300 mg/day
Ionized calcium	1.0 mmol/L	1.12–1.32 mmol/L	1.15 mmol/L

## Discussion

Hypocalcemia is an electrolyte imbalance that may exhibit a range of clinical symptoms, from being asymptomatic when mild to causing life-threatening seizures, persistent heart failure, or laryngospasm when severe [7].

The hallmark of acute hypocalcemia is neuromuscular irritability. Patients often complain of numbness and tingling in their fingertips, toes, and the perioral region. Paresthesias of the extremities may occur, along with fatigue and anxiety. Muscle cramps can be very painful and progress to carpal spasm or tetany.

When the adjusted total serum calcium level dropped below 7.0 mg/dL or the ionized calcium level was less than 1.1 mmol/L, tetany was usually observed.

Neuromuscular​‍​‌‍​‍‌​‍​‌‍​‍‌ irritability leading to tetany is typical of the acute hypocalcemia. The symptoms may vary from minor, such as paresthesia, to extreme ones, e.g., carpopedal spasms, laryngospasm, and convulsions [8].

Intense vomiting can also lead to metabolic alkalosis and a decrease in ionized calcium levels which may induce tetany and carpopedal spasms [9].

Trousseau’s sign comes about when a blood pressure cuff is inflated to 20 mm Hg above the patient’s systolic level for 3–5 min. Wrist flexion with extension of interphalangeal joints and the thumb being abducted is when the carpal spasm occurs. The spasm can be very painful [10].

Proton pump inhibitors inhibit the gastric H+/K+-ATPase by covalently connecting to cysteine residues of the proton pump. Every proton pump inhibitor should be in the parietal cell with acid accumulation via protonation, then the activation is facilitated by the second protonation at the active secretory canaliculus of the parietal cell [11]. Clinical trials suggest that PPI-induced hypochlorhydria results in a decrease in calcium absorption in the small intestine that eventually causes blood calcium to lower ‍​‌‍​‍‌​‍​‌‍​‍‌[12]. Acute hypocalcemia may have cardiac manifestations. Prolongation of the QT-interval due to lengthening of the ST-segment on electrocardiogram is fairly common in hypocalcemic patients. T-waves are abnormal in approximately 50% of patients [13]. Changes in smooth muscle function with low serum levels of calcium may cause irritability of the autonomic ganglia and can result in dysphagia, abdominal pain, biliary colic, wheezing, and dyspnea.

An important biochemical feature in this case was the inappropriately low PTH level in the setting of severe hypocalcemia, where a compensatory elevation would ordinarily be expected. This raises the possibility of a transient impaired parathyroid response. Although serum magnesium was not frankly low, it was within the lower-normal range, and subtle magnesium-related impairment of PTH physiology cannot be fully excluded. The subsequent rise in PTH at discharge suggests recovery of the parathyroid axis.

Serum magnesium was normal, but subtle effects on PTH cannot be excluded. The rise of PTH to 50 pg/mL at discharge supports recovery, and while chronic PPI use may reduce calcium absorption, additional transient parathyroid dysfunction likely contributed.

Magnesium in the intestine is absorbed via both a passive and an active transport system, which is dependent on two proteins situated on the enterocytes’ apical membrane, the transient receptor potential melastatin TRPM6 and TRPM7. These proteins are highly selective for magnesium absorption and are involved in the regulation of magnesium levels during times of low dietary magnesium intake [14]. The activity of TRPM is influenced by the acid, base condition of the intra-luminal environment in which an acidic medium increases its activity [15]. PPIs reduce the activity of TRPM6 leading to less absorption of magnesium from the intestine and hypomagnesemia [15].

Several previous reports have described electrolyte abnormalities associated with prolonged PPI use. Most cases involve hypomagnesemia leading to secondary hypocalcemia, but rare cases of severe hypocalcemia without significant hypomagnesemia have also been reported [16–18]. These reports support the plausibility of PPI-induced mineral imbalances and emphasize that clinicians should consider this possibility in patients presenting with unexplained hypocalcemia.

## Conclusion

Long-term proton pump inhibitor therapy can lead to clinically significant hypocalcemia, even in otherwise healthy individuals, manifesting as acute neuromuscular irritability, carpopedal spasms, and ECG changes. Prompt recognition, discontinuation of the PPI, and timely calcium replacement result in rapid symptom resolution and normalization of laboratory values. Clinicians should include PPI-induced hypocalcemia in the differential diagnosis of unexplained tetany and maintain follow-up of serum calcium and parathyroid function to ensure sustained recovery and prevent recurrence.

## Data Availability

No datasets were generated or analyzed during the current study.
